# The Crime of Administrative Blindness

**Published:** 1912-11-30

**Authors:** 


### HOSPITAL WEAKNESS AND HOSPITAL STRENGTH.

(Contributions to this page will be welcomed.)

### The Crime of Administrative Blindness.

Every now and then even the greatest of public institutions, not merely the smaller and less important ones, is cursed with an atmosphere of inefficiency, it may even be of wrongdoing and injustice on the part of officials highly placed, who from various causes, though once highly competent, may have become a danger and not a strength to the reputation and popularity of an institution. If we were to say that in a great hospital, for instance, well known, not only in London but all over the world, the gradual and increasing tyranny and injustice of a high official has caused the great body of its staff to be in a state of rebellion, it is probable that few would credit the truth of such an assertion. Yet, as a matter of fact, we are assured that a strike is being spoken of with increasing vehemence and insistence within the walls of one of our great hospitals at the present time, where, so far as we can make out, the staff have too much reason for their dissatisfaction and discontent. Again, who would credit if we were to state that the head of a great nursing organisation has gradually wrapped herself up within herself to an extent which makes her disregard most of the principles of justice? This high official it is maintained does not hesitate in her reports to make statements which the facts do not justify. In these days, where hospitals of the best type insist upon a standard of education for the nursing staff, it follows that liberties of this kind cannot be taken with the sisters and nurses with impunity, and that sooner or later the officer at fault must be brought to book, and be pensioned off or speedily removed from office. Is there a matron who is worthy to hold a high position in a hospital, who would attempt to justify the retention of a certificated nurse on night duty for many months together, or to restrict her to the work of cutting bread and butter, whilst few or no opportunities are offered where she can take her part in the skilled nursing and attendance of the sick? Similarly, the retention of nurses on night duty for a longer period than three months, as a maximum, is not a justifiable course. Wherever this abuse exists, especially in those cases where some nurses are retained on night duty for many months at a time, the matron responsible for such an exhibition of want of care for the health and welfare of her nurses should be made by the chairman or treasurer to feel her incapacity for such a position.

It may be asked what have these matters to do with the crime of Administrative Blindness? Our answer is everything. Why? Because no such things could occur if the chief honorary official, especially if he be a resident, had his eyes open and was free from prejudice. It is the plain duty of every such official to insist upon knowing and seeing and justly assessing everything that is happening within the walls of his hospital. No one can more strongly advocate consistently than we have done in these columns the duty of supporting the higher officials of a hospital in the difficult task of administering the great departments under their charge. But this attitude on our part entitles us to claim that Administrative Blindness which fails to prevent cruelties, injustice, dangerous risks to health for the humbler members of any department of a hospital, through the inefficiency of one of the principal officers, should make the chairman or treasurer or superintendent recognise that either he should wake up and do his plain duty, or make way by retirement for a real live man of sufficient capacity to prevent these things. In no other way can he save the hospital from the disaster with which it may be threatened by the continuance of proceedings such as those we have indicated, without attempting to describe, at present, the actual condition of inefficiency of a high official, of which they are so clear an indication.

Only the other day, in course of conversation, we heard it said that the son of the head of one of our great hospitals was complaining bitterly of the injustices perpetrated within its walls. When he was asked why he did not point these things out to his father and urge him to do his duty, his reply was: My father is too obstinate and too old to wake up or to suffer any remonstrance however justifiable. It is a truth which is becoming more and more widely recognised and acted upon by hospital administrators all over the world, in the present day, that when officials, however highly placed, exhibit idiosyncrasies, such as those we have been considering, the time has come for their retirement. If they are stipendiaries and have done years of service, then they should be pensioned off without a moment's delay or hesitation; if they are voluntary workers, then pressure should be brought to bear upon them to make them take the well-earned retirement, which is the reward of us all, when we have attained a certain age. We hope, at any rate, that what we have here written of knowledge, with a strong sense of responsibility, may not fail to reach the notice of all such perpetrators and defaulters. If they do, the result may be speedy, and should prove wholly to the advantage of all concerned. No great hospital, however financially strong, can maintain its reputation, or do its work properly, whilst it is afflicted with the presence of an official as honorary chief administrator who is guilty of conduct such as we have called attention to in the course of this foreword and remonstrance.

